# Measuring network rewiring over time

**DOI:** 10.1371/journal.pone.0220295

**Published:** 2019-07-24

**Authors:** Yicheol Han, Stephan J. Goetz

**Affiliations:** 1 Department of Agricultural and Rural Policy Research, Korea Rural Economic Institute, Naju-si, Jeollanam-do, Republic of Korea; 2 Department of Agricultural Economics, Sociology, and Education, Pennsylvania State University, University Park, Pennsylvania, United States of America; 3 Northeast Regional Center for Rural Development, Pennsylvania State University, University Park, Pennsylvania, United States of America; Universidad Rey Juan Carlos, SPAIN

## Abstract

Recent years have seen tremendous advances in the scientific study of networks, as more and larger data sets of relationships among nodes have become available in many different fields. This has led to pathbreaking discoveries of near-universal network behavior over time, including the principle of preferential attachment and the emergence of scaling in complex networks. Missing from the set of network analysis methods to date is a measure that describes for each node how its relationship (or links) with other nodes changes from one period to the next. Conventional measures of network change for the most part show how the degrees of a node change; these are *scalar comparisons*. Our contribution is to use, for the first time, the cosine similarity to capture not just the change in degrees of a node but its relationship to other nodes. These are *vector (or matrix)-based comparisons*, rather than scalar, and we refer to them as “rewiring” coefficients. We apply this measure to three different networks over time to show the differences in the two types of measures. In general, bigger increases in our rewiring measure are associated with larger increases in network density, but this is not always the case.

## Introduction

Interest in network analyses has exploded in recent years across diverse disciplines, ranging from the physical [1] to the bioecological [2, 3] and social sciences [4]. The growing interest is in part due to increased availability of *big data* from social media or on-line searches. Fraiberger et al. [5], for example, use data on networks of common exhibitions from Magnus (www.magnus.net) to examine how artists’ initial ranking affects their lifetime success. The interest is also explained by the near-universal applicability of new tools and insights developed, with perhaps two of the most important studies being on the dynamics of small world networks [6] and the emergence of scaling in self-organizing systems [1].

As more observations become available on the same networks over time, the question of network changes and dynamics across diverse data sets has become more important. These include formally analyzing how networks change, or “rewire” over time. Dynamic network studies focus on issues *within* a given network, such as the attachment method [1, 7], network failure [8], or community structure [9]. In contrast, no prior study has used the cosine similarity to show how relationships of nodes within the same network change to all other nodes from one period to the next.

Also of interest in this context is understanding not only the determinants but also the impacts of network rewiring on different network performance indicators. For example, the amount of rewiring in a regional commuting network may depend on local labor market flexibility. Such rewiring may in turn have different effects on the amount of local traffic congestion. In the case of a scientist collaboration network, a greater degree of rewiring may affect future success or productivity of the scientists within the network. Or, junior scientists may benefit more from changing their patterns of collaboration than senior scientists. Thus, it is important to be able to measure changes both for individual nodes as well as for the entire network.

This article proposes a straightforward measure for comparing the same network at two different points in time. In particular, we use the cosine distance or dissimilarity function to develop new insights into the changes or rewiring of various networks. The use of this measure is to the best of our knowledge unprecedented in the network literature. Following Albert et al. [8] we use the term “rewiring” to refer to changes in the network. This primarily suggests changes in the links between nodes. We apply the proposed measure to the cases of an economic input-output table viewed as a network, a commuting network, and a network of scientist-collaborators.

## Rewiring in networks: Method

### Absolute rewiring

Consider generic network ***N***, consisting of linked nodes which can be represented in the form of a stacked vector. Two networks thus can be compared using the angle between them, defined as the cosine distance. The distance between networks **A** and **B**, *δ*, is calculated as:

δ(A,B)=1−Θ(A,B) (1)

where cosine similarity function Θ between **A** and **B** is calculated as:

$$\Theta (A,B)=\frac{J(A\circ B)J^{T}}{{\left\|A\right\|}_{F}{\left\|B\right\|}_{F}}=\cos\theta_{\mathrm{AB}}$$ (2)

Here **J** is a 1×*n* vector where each element is one, and **J**^T^ is its transposed vector. Operator ∘ is the Hadamard product, ||**A**||_F_ the Frobenius norm of matrix **A**, and *θ*_**AB**_ the angle between **A** and **B**. If **B** is network **A**_*t’*_ measured one time step later (**B** = **A**_*t’*_), then the cosine distance function *δ*(**A**_*t*_, **A**_*t’*_) indicates the degree of change or *absolute rewiring coefficient* of network **A** over time.

It can be seen that 0 ≤ *δ* ≤ 1, which allows us to compare the rewiring across different networks, even those of different sizes. For example, a network with *δ =* 0.5 exhibits a greater degree of rewiring (or reorganization of the relationships [links] among its nodes) than a network for which *δ =* 0.3.

### Relative rewiring

While the rewiring coefficient *δ* is intuitively appealing as a measure of change in the network (or matrix) representation, it does not convey a *direction* of change. In two-dimensional space the new vector may lie above or below the original vector, while pointing in the same or the opposite distance. To assess the direction of change, we can compare the network change to the equivalent, same-period change in some reference or benchmark matrix, **A**^*R*^. For example, we may compare how one industry changed relative to changes in the overall economy, or to another industry vector. Last, we may examine how the commuting patterns (vectors) of one county or metropolitan statistical area (MSA) changed compared to counties elsewhere.

To measure this directional change we propose a *relative rewiring coefficient δ*^*R*^ (relative to some benchmark or reference network) as follows:

$$\delta^{R}=\Theta (A_{{}^{t}},A_{t}^{R})-\Theta (A_{{}^{t'}},A_{t'}^{R})$$ (3)

It can be seen that –1 ≤ *δ*^*R*^ ≤ 1. In this case a large positive value of *δ*^*R*^ indicates that the network has become *more* similar to the benchmark or reference economy. A smaller value indicates that the network has become more dissimilar from the benchmark economy. Below we show examples of these. In addition to the above, we calculate and report various network centrality measures to show how relationships among nodes changed during the rewiring process. In particular, networks may become more or less dense over time. We compare these changes in network density with results obtained using the cosine similarity measure.

The next section shows both absolute (*δ*) and relative rewiring (*δ*^*R*^) coefficients over time for different types of networks, and also rewiring of a subnetwork within the network. In this case we consider the U.S. economy as part of the global economy.

## Applications

We apply the cosine distance rewiring measure to three representative networks for which we have data at multiple points in time. We start by analyzing the economic input-output (IO) table as a network. While network studies of the IO table are not new (see, e.g., [10–23]), prior studies have not focused on the rewiring of the IO table over time, or on how to measure or model this rewiring.

### Rewiring of the world IO table

**Fig 1:**
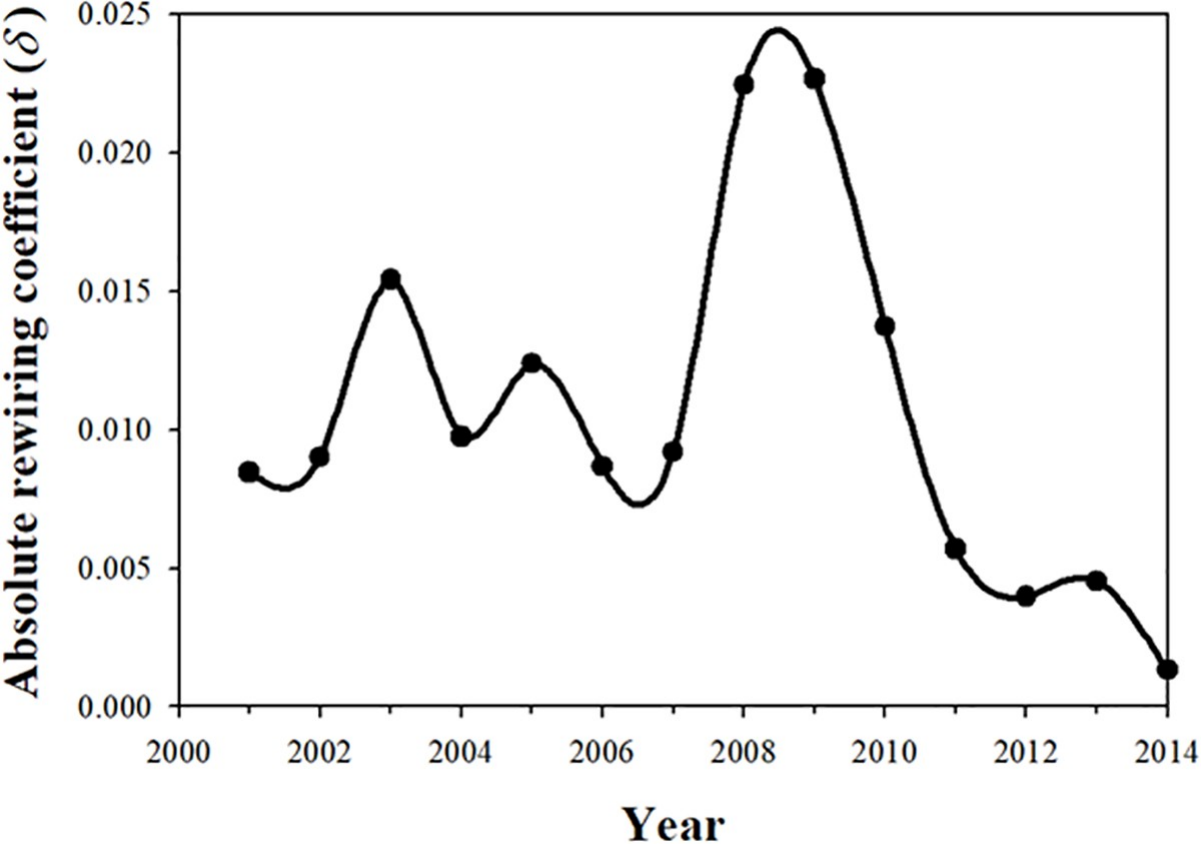
Year-to-year rewiring coefficient (*δ*) of the world IO network, 2000–2014. Data source is the World IO table.

Fig 1 shows year-to-year absolute rewiring coefficients in the world IO table from 2000 to 2014. A spike in rewiring is evident in 2008 and 2009, which is expected because of the Global Financial Crisis (GFC). Even in the years leading up to the GFC, fluctuations occurred in the amount of rewiring, but all are smaller than those observed in 2008 and 2009. Furthermore, the pronounced downward trend in rewiring after 2010 compared with the pre-GFC rewiring is notable. One hypothesis is that this lack of rewiring contributed to the lackluster global economy in the post-GFC period.

Here we have included intermediate transactions both within each nation’s economy and transactions across countries, i.e., flows representing imports and exports. This analysis can also easily be done separately for individual nations without imports and exports, if the internal workings of an economy are of primary interest.

**Fig 2:**
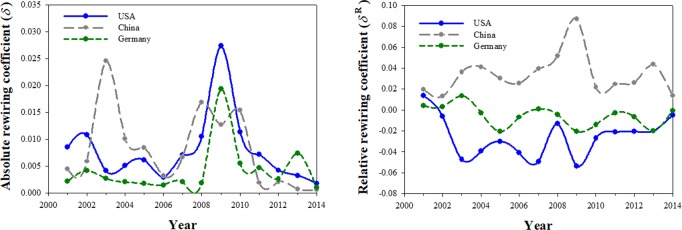
**Absolute (*δ*, left panel) and relative rewiring coefficients (*δ***^***R***^**, right panel) of the U.S., Chinese, and German economies, 2000–2014.** Data source is the World IO table.

**Fig 3:**
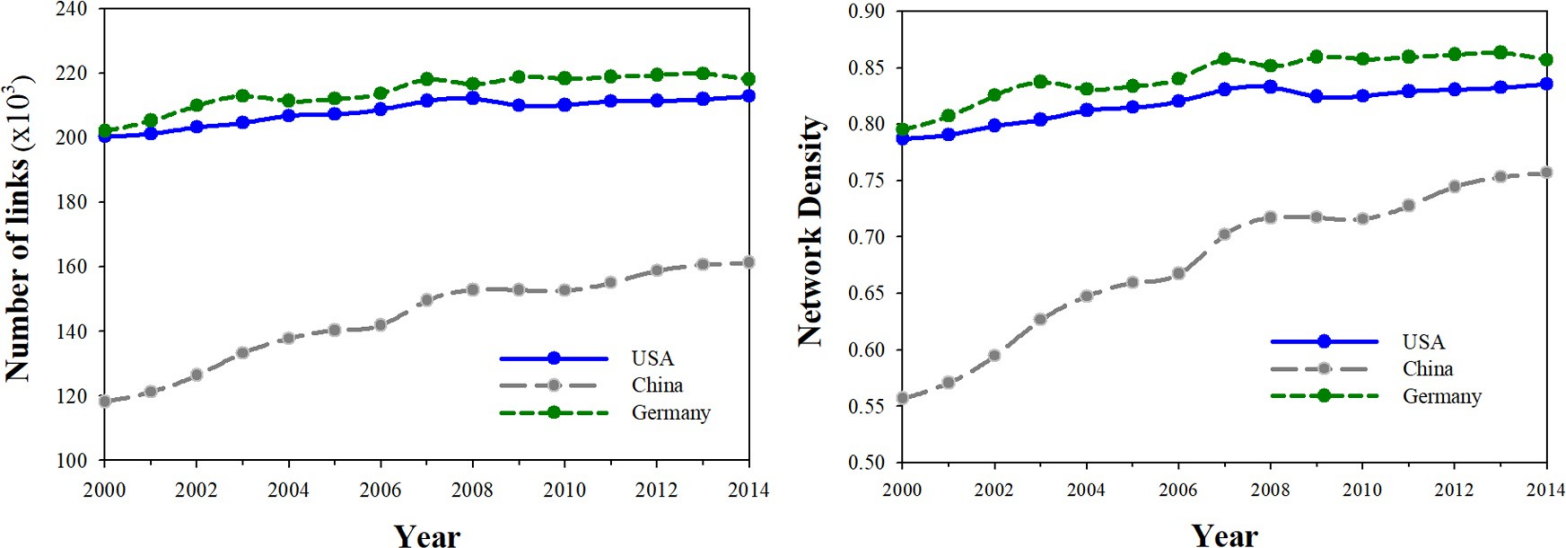
**Number of links (left panel) and network density (right panel) of the U.S., Chinese, and German economies, 2000–2014**. Network density is estimated as a ratio of the number of actual links to the number of potential links in the network. Data source is the World IO table.

Next we examine the year-to-year absolute rewiring patterns over the same time period for three representative countries (USA, Germany, and China; see Fig 2 left panel). Significantly, the impact on China of joining the World Trade Organization (WTO) in 2003 was larger than the impact of the GFC on that nation (*δ* = 0.025 in 2003 vs. 0.013 in 2009). We note below that this fact is not captured by the conventional measure of network change, that is, either the number of links or the network density (c.f. Fig 3). Our proposed measure efficiently shows the relative sizes of these twin shocks on China's macroeconomy, as measured by its IO Table. Rewiring in China in 2009 also was smaller than in 2008 or 2010, and it was smaller than in either Germany or the U.S. In both the U.S. (*δ* = 0.027) and in Germany (0.019)–again, as representative examples–the peak rewiring occurred in 2009 rather than 2008. In terms of the amount of rewiring, the U.S. was the most affected by the GFC of the three economies shown. The amount of rewiring tapered off for all economies post-GFC, with Germany showing a small one-year increase in 2013.

In Fig 2 (right panel) we plot the relative rewiring coefficient for each of the countries of primary interest. Here the comparison is between how the nation rewired relative to the global economy. China experienced the largest *relative* rewiring during the GFC (*δ*^*R*^ = 0.087) rather than during the period of its accession to the WTO (*δ*^*R*^ = 0.041). The fact that *δ*^*R*^ >0 implies that the Chinese economy became more like the global economy in this period. Interestingly, it did so more in this period than in 2003, when it joined the WTO. Thus the absolute and relative rewiring coefficients are picking up subtle effects, and they do not always move in the same direction. The U.S. and to a lesser extent Germany generally show *δ*^*R*^ <0, implying that they rewired in a direction different to the rewiring of the global economy. In this period the rewiring of the U.S. economy away from the global economy was greater than was the case for Germany.

The rewiring that occurs within individual nations may be the result of changes in production technology, changes in import or export relationships with other countries, or other factors. With our measure, we are not able to ascertain the source of these changes; doing so would require a structural decomposition analysis. Our method does not allow for or provide such an analysis, but this would shed further light on whether any observed changes are due to shifts in final depend components or changes in the elements of the IO table, for example. More specifically, Blair and Miller [24] (p. 593) discuss the decomposing of a change in the IO table from one period to the next into components such as technological change and demand change, or even more subtle changes in factors such as energy use, value added, employment, etc. Our paper focuses only on overall change between elements of the IO table, and is not able to distinguish between these more nuanced sources of change.

We next compare the new results with conventional measures of network change. We apply these to the U.S., German and Chinese economies. Fig 3 shows the number links (left panel) and density of links (right panel) as conventional network-based measures of rewiring (or network change) within these economies during 2000–2014. As the more mature and developed nations in this sample, the U.S. and Germany unsurprisingly had more links than China. Certain industries present in the other two nations do not exist in China. However, China is catching up over this period, with the number of links rising by 33% from 120,000 to 160,000.

The increasing network density (Fig 3, right panel) represents a maturing of the economy as transactions between new and existing industries expand. The network density of inter-industry transactions rose substantially over this period in China, but it is still less than for the U.S. or Germany. The U.S. and German economies show similar patterns of network density.

**Fig 4:**
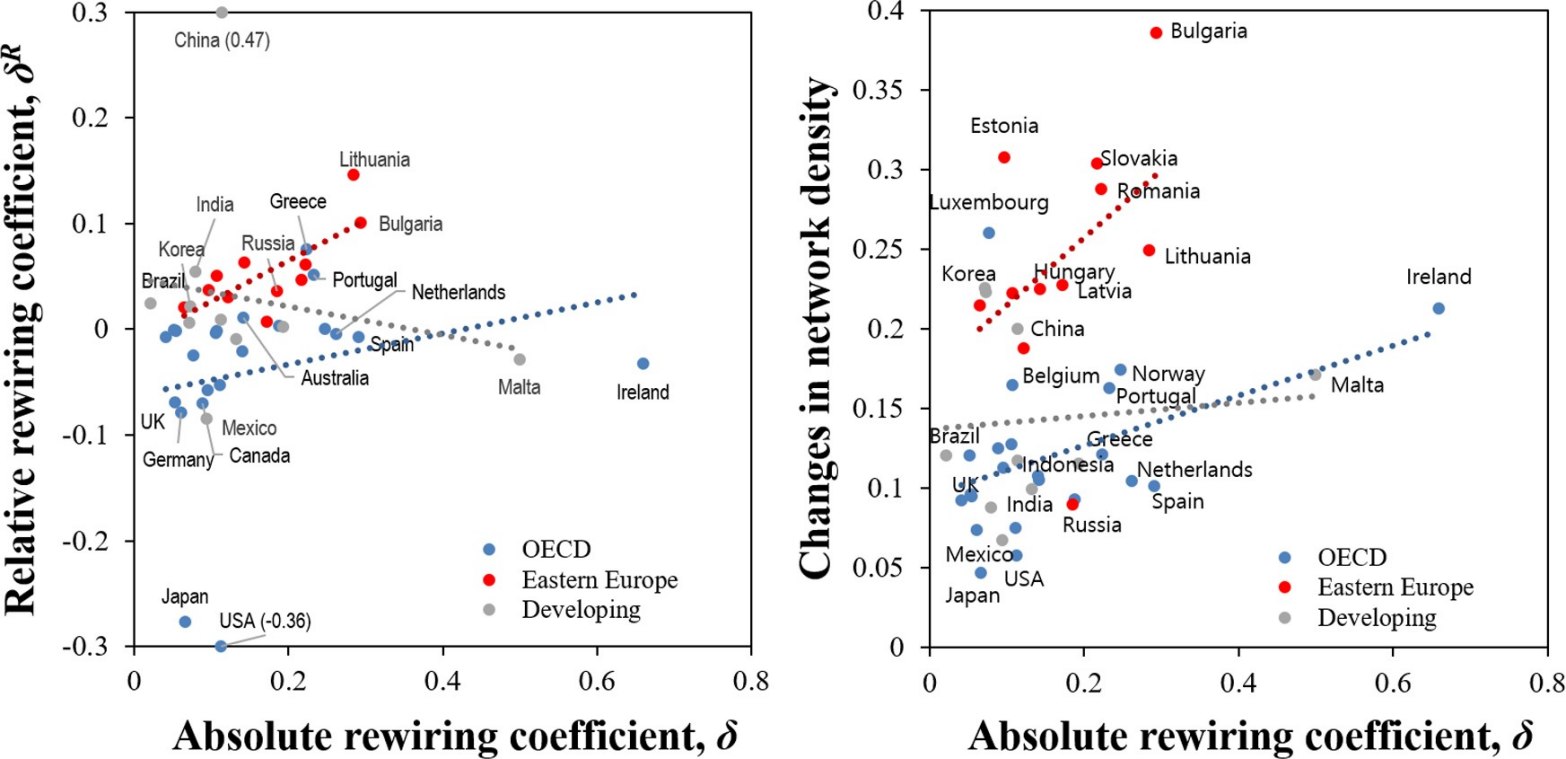
Relative vs. Absolute rewiring coefficients, 43 countries, 2000 to 2014. Early OECD member countries before 1990 (Blue) and Eastern Europe countries (Red) are highlighted. Other developing nations shown in gray.

More insights may be gained by plotting the absolute rewiring of a nation’s economy against the relative amount. Fig 4 (left panel) shows that Ireland (especially), Malta, Spain and The Netherlands each rewired to different absolute degrees over the period 2000–2014, but had similar relative rewiring coefficients (*δ*^*R*^ ≈0). In a network sense they remained similar to the global economy. Conversely, China, India, Japan and the U.S. each had small absolute rewiring coefficients, but different relative rewiring coefficients. As rapidly developing nations, China and India are becoming more like the global economy (*δ*^*R*^ ≫0). In the U.S. and Japanese economies the economic rewiring is over time differentiating them further from the global economy (*δ*^*R*^ ≪0).

Excluding Japan and the US, Eastern European countries had a similar range of relative rewiring coefficients compared to the OECD countries. In general their relative rewiring followed the world economy, with *δ*^*R*^ *>*0. The OECD countries tended to rewire in a direction that was different from that of the global economy (*δ*^*R*^ *<*0). For these countries, more absolute rewiring was generally associated with more relative rewiring. For the other developing countries the opposite relationship is evident. Here there is a weak inverse relationship between absolute and relative rewiring. We also note finally, the high R2-square statistic on the relationship between absolute and relative rewiring in the case of the Eastern European nations.

Perhaps most importantly, the left hand panel of Fig 4 shows key differences between conventional density measures and our proposed measure of network change. For Eastern European countries especially, more rewiring is associated with more change in density. But for the the gray-colored (developing) countries, a starkly different pattern emerges. Here our measure suggests considerable network change whereas the conventional density measure does not. We conclude that conventional measures such as network degrees and density may suggest important network change where in fact none exists, as revealed by the cosine similarity measure. The opposite is also true, and this finding warrants further investigation.

Next we are interested in examining how this rewiring may have affected the economic growth rates of different countries. To do this we statistically estimate a parsimonious, conventional economic growth model that relates our rewiring measures to the compound annual growth rate in GDP/capita (Δ*GDPcap*), as a measure of aggregate behavior, while holding constant initial (year 2000) GDP/capita in the starting period (*GDPcap*_0_), population density (*PopDen*_0_) as a proxy for agglomeration, as well as country fixed effects (*CFE*) to capture level of development or integration. This produces the following estimating equation, with *α*_*i*_ denoting coefficients to be estimated:

$$\Delta GDPcap=\alpha_{0}+\alpha_{1}GDPcap_{0}+\alpha_{2}PopDen_{0}+\alpha_{3}\delta +\alpha_{4}\delta^{R}+CFE$$ (4)

**Fig 5:**
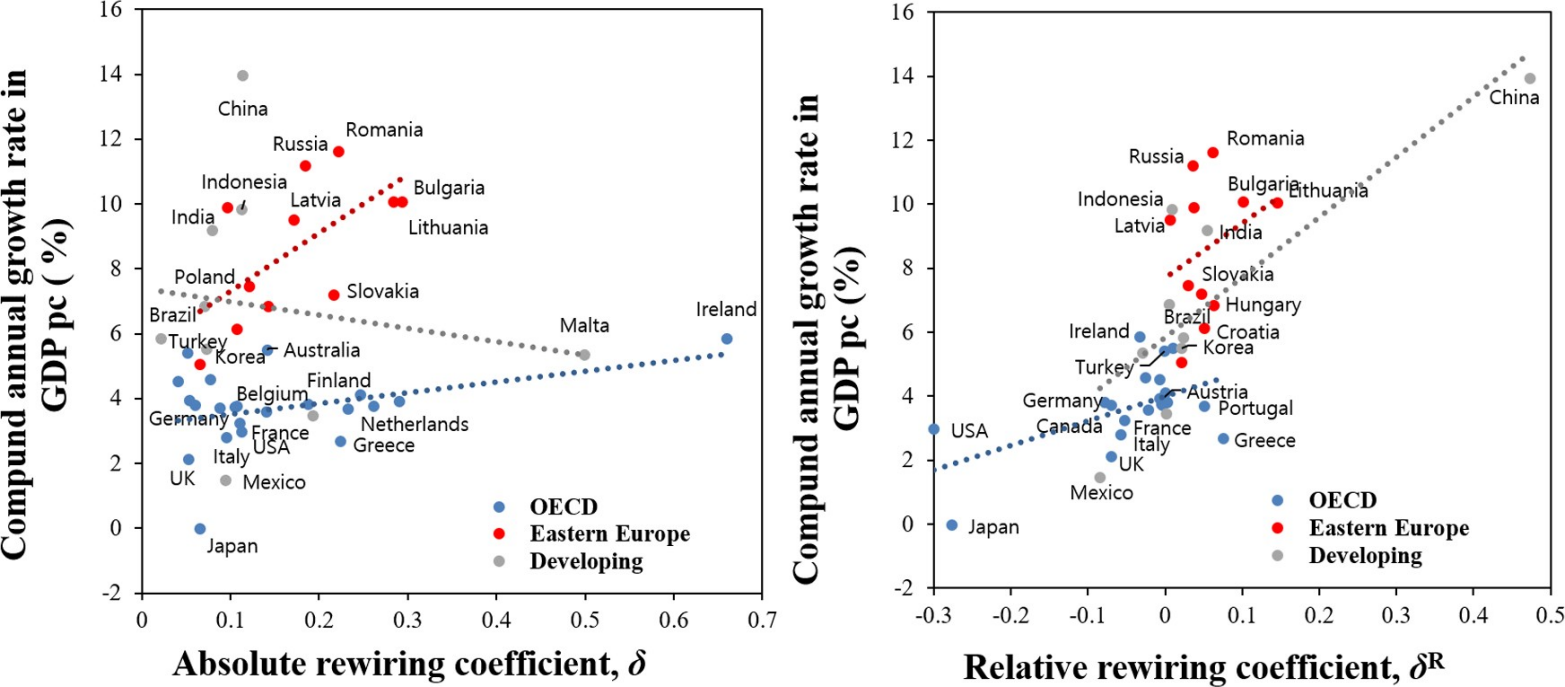
Plot of the relationship between the rewired measures and compound annual growth rate in GDP/capita for 42 countries. Early OECD member countries before 1990 (Blue) and Eastern Europe countries (Red) are highlighted. Other developing nations shown in gray. GDP data are in constant 2010 U.S. dollars from the World Bank.

*GDPcap*_0_ captures a range of economic growth preconditions, such as education. When we do this, the calculated absolute and relative rewiring measures do independently and jointly have statistically significant associations with the compound annual growth rate (see Table 1; Fig 5). Thus, the more each country rewired in either an absolute or relative sense, or both, between 2000 and 2014, the more it also enjoyed faster per capita GDP growth over the period 2000–2017 (allowing for a small time lag relative to when the rewiring is measured).

**Table 1:** Regression results for GDP per capita growth rate and rewiring measures.

	Basic	Absolute only	Relative only	Absolute & Relative
Initial GDP pc, log	-0.771***	-0.779***	-0.566***	-0.582***
	(-4.63)	(-4.71)	(-5.07)	(-5.15)
Population density, log	-0.08	-0.087	-0.080	-0.085
	(-1.17)	(-1.37)	(-1.47)	(-1.65)
Absolute rewiring		0.132**		0.096*
		(2.50)		(1.70)
Relative rewiring			0.329***	0.312***
			(3.19)	(2.84)
_cons	***	***	***	***
	(5.81)	(5.87)	(6.57)	(6.77)
Countries group Fixed Effect	Yes	Yes	Yes	Yes
N	42	42	42	42
Adjusted R2	0.7182	0.7299	0.7918	0.7964

Dependent variable is the compound annual per capita GDP growth rate over the period 2000–2017. The table shows standardized coefficients (beta) with robust standard errors and *t*-statistics in parentheses. Significance levels: different from zero at *10%, **5%, and ***1% or lower. Countries Group Fixed Effect: country membership in OECD, Eastern Europe or Developing group.

### Rewiring of a commuting network

Next we apply the cosine similarity function to a commuting network, using U.S. Census data. The U.S. county-to-county commuter flows are links, and counties represent nodes. Each county *i* ∈ *I* receives commuters from or sends commuters to another set (vector) of counties. This creates a matrix of dimension *IxI*, with data for three points in time: 1990, 2000 and 2010. We use these to calculate rewiring over two distinct time periods.

**Fig 6:**
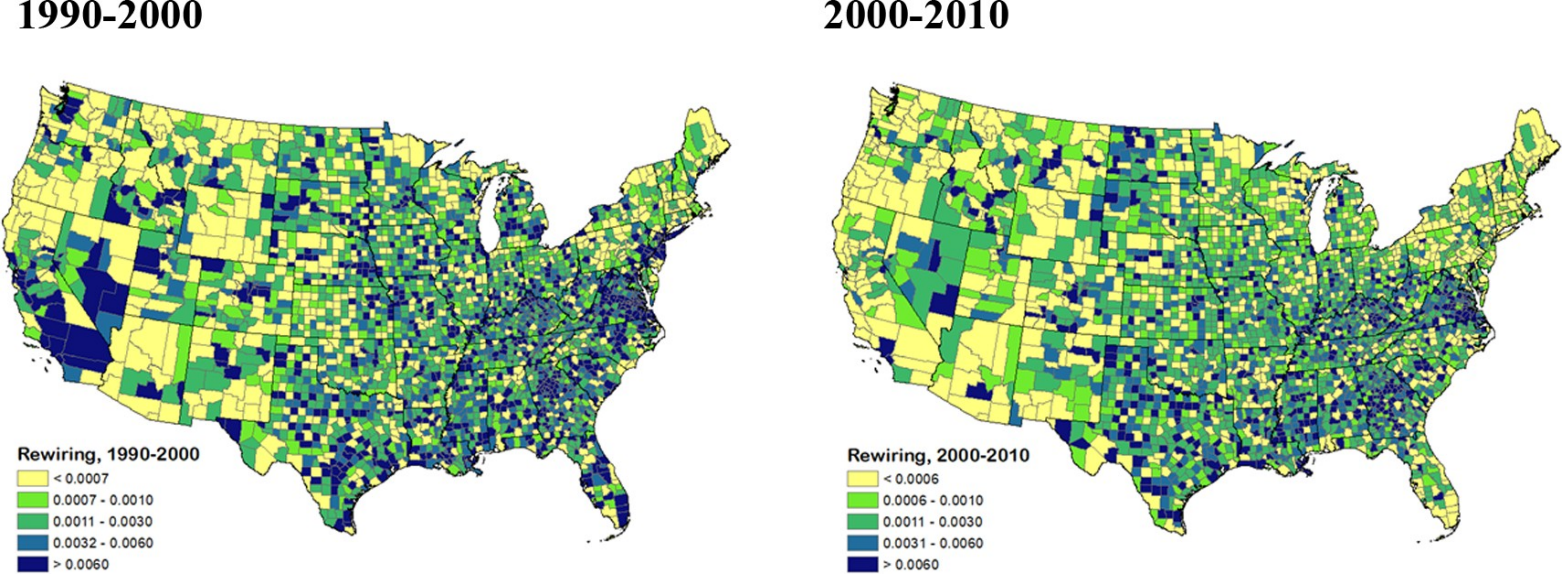
Commuting network absolute rewiring, U.S. counties, 1990–2000 and 2000–2010.

Fig 6 maps absolute rewiring coefficients for 3,108 U.S. counties over two time periods. In the panel covering 1990–2000, commuting networks in some metro areas and states showed substantial rewiring, with *δ* >0.006. The New York City-Philadelphia-DC metro areas saw substantial changes, which continued on into Virginia (including Fairfax, Richmond and Norfolk) as well the Research Triangle area of North Carolina. This rewiring likely reflects ongoing suburbanization or urban sprawl, as homeowners increasingly purchased larger homes with yards. The seeds of the GFC also had their origins in this early period. These developments were not limited to the eastern seaboard, however.

Other areas with substantial commuter network rewiring over the period 1990–2000 can be seen in South Carolina, especially around Atlanta, Georgia as well as Orlando and Miami, Florida and on into San Antonio and Austin, Texas. Denver, Colorado, southern Nevada (one of the epicenters of the GFC in the U.S.), southern California and Seattle, Washington, North Dakota, Michigan and Ohio also had county clusters experiencing significant rewiring.

Comparing the two different periods, more commuting network rewiring occurred in Washington, California, Michigan and especially the New York City area as well as Florida in the 1990–2000 period compared to 2000–2010. To some extent this reflects reductions in economic activity and associated commuting caused by the GFC, which had more severe impacts along the coasts. It could also mean that less rewiring occurred over time as adjustments in commuting patterns as well as associated housing development matured and levelled off, for example, around Atlanta, Georgia. Some greater degree of rewiring in commuting networks is evident in North Dakota, likely reflecting early activity associated with shale gas exploration and subsequent development. Although we do not present the results here, we can also calculate relative rewiring coefficients for each individual county, relative to some reference region. For example, this could be the entire nation, or one of the Census subregions, such as the Northeast.

**Fig 7:**
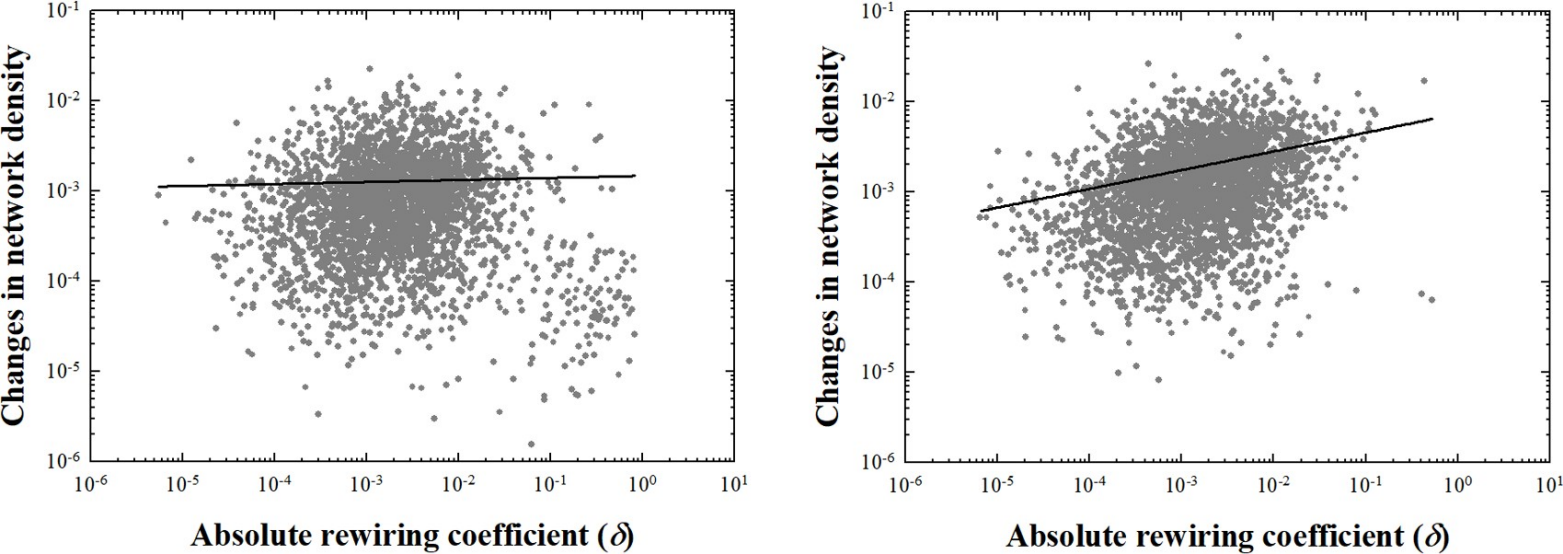
**Relationship between absolute rewiring coefficients and changes in density in commuting networks, 1990–2000 (left panel) and 2000–2010 (right panel).** Density changes are measured by subtracting density in *t*+1 from density in *t*.

Fig 7 plots changes in network density vs. the absolute rewiring coefficients over two time periods for the commuting network. The 1990–2000 (right hand) panel shows clearly that more rewiring also is associated with larger changes in structural properties as measured by the change in network density. In future work it would be of interest to assess whether this relationship also exists in different parts of the U.S. and for different county types, such as those that are urban, suburban or rural. The 2000–2010 panel reveals a similar relationship, although the tighter clustering of coefficients and changes makes this more difficult to assess.

### Rewiring of a scientist collaboration network

Our final application is to a network of scientist-collaborators, compiled by the authors, with changes measured over the years 2006, 2010 and 2012. Table 2 shows summary statistics for the nodes making up the network. The scientists represent four different disciplines; three individuals (Others) are not thus classified. Binary ties among the scientists are measured as collaborations (on grants, papers or phone calls) over the course of the preceding year. The simple illustration presented here provides a different perspective on collaboration behavior than, for example, the seminal work of Newman [25]. Recent work in this area includes Haiyan et al. [26] and the more conventional type of network analysis reported in Harris et al. [27]. This could also be replicated with the data used here, although the sample size is smaller. Our analysis provides a different perspective on the degree to which scientists from different disciplines collaborate with one another.

**Table 2:** Summary statistics for the scientist-collaboration network.

Discipline	#nodes	Gender (M/F)	Avg. links 2006	Avg. links 2010	Avg. links 2012
Discipline1	4	4/0	4.0	18.8	34.5
Discipline2	2	2/0	2.0	14.0	36.0
Discipline3	5	4/1	3.8	12.0	29.6
Discipline4	4	0/4	7.5	14.8	35.0
Others	3	0/3	8.3	16.7	35.7

Note: M = males, F = females

**Fig 8:**
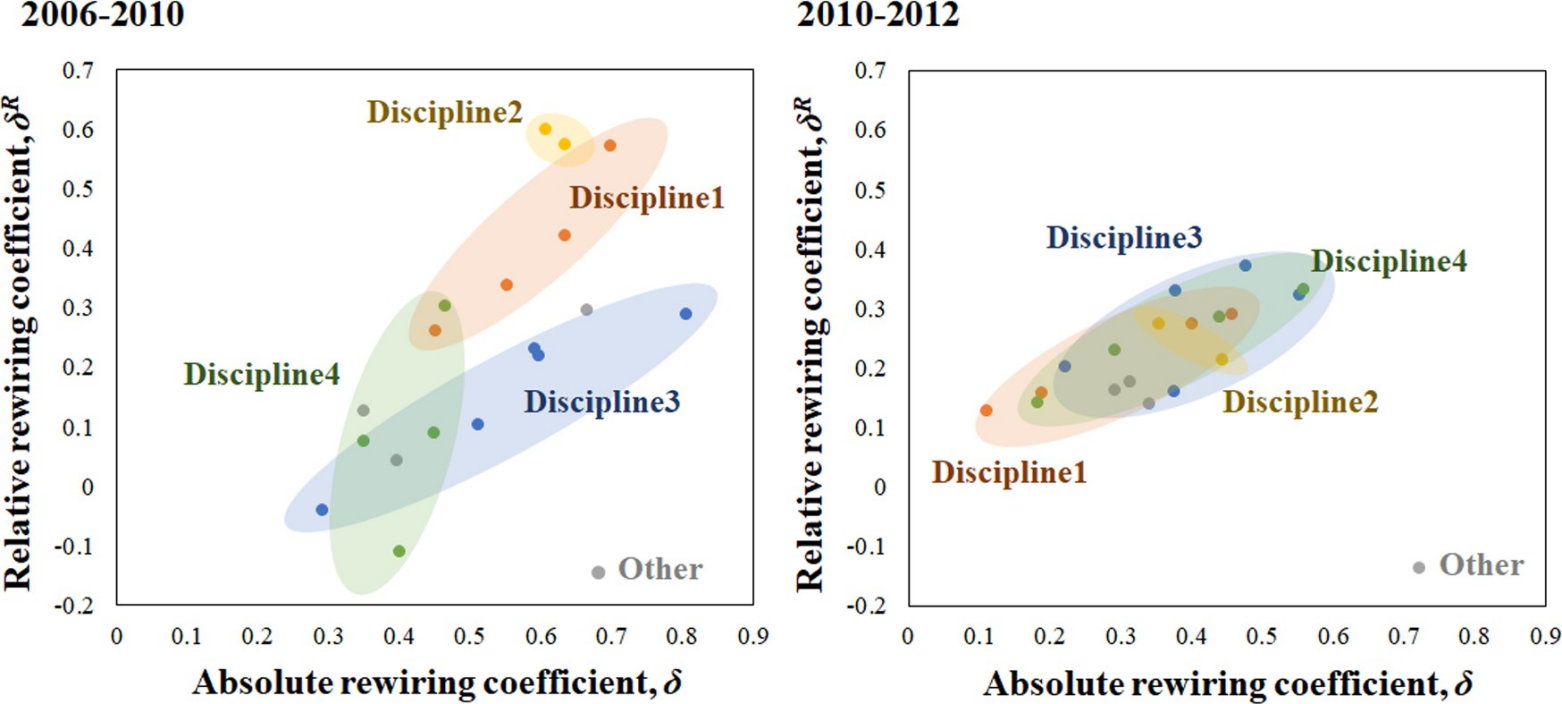
Absolute and relative rewiring in a scientist collaboration network, 2006–2010 (left panel) and 2010–2012 (right panel).

Scientists from the same disciplines tend to cluster in terms of changes in their collaboration behavior (Fig 8). This is shown by the color shading. For example, in the period 2006–2010 scientists in Disciplines 1 and 2 showed the greatest amount of absolute and relative rewiring compared to the other disciplines shown. This means that these scientists individually and collectively changed their connections to other scientists in the network the most. They also became more like the other scientists in terms of their connections or collaboration patterns with other network members.

Conversely, those in Discipline 4 showed the smallest absolute and relative rewiring coefficients. Scientists in Discipline 3 had more variation in absolute rewiring, and a relatively smaller amount of relative rewiring. However, in the period 2010–2012, scientists in all disciplines (except Discipline 2) follow nearly identical absolute and relative rewiring patterns (Fig 8, right panel).

In general, Discipline 3 scientists rewired their networks the most, but had a lower degree of rewiring than those in Disciplines 1 and 2. As noted, the latter group showed the largest degrees of relative rewiring, becoming more like the overall network, with Discipline 1 showing more absolute rewiring than Discipline 2 scientists. Overall, a remarkable shrinking *and* overlapping or merging of the shaded areas can be seen in the two panels, suggesting that the collaboration networks are becoming more similar over time, as the scientists from different disciplines become more comfortable working with one another. The size of the relatively small network also constrains the number of *new* relationships (ties) that can be formed, but the overall pattern may also signal collaboration fatigue, which could be investigated in future research by examining the productivity of individual scientists over time.

**Fig 9:**
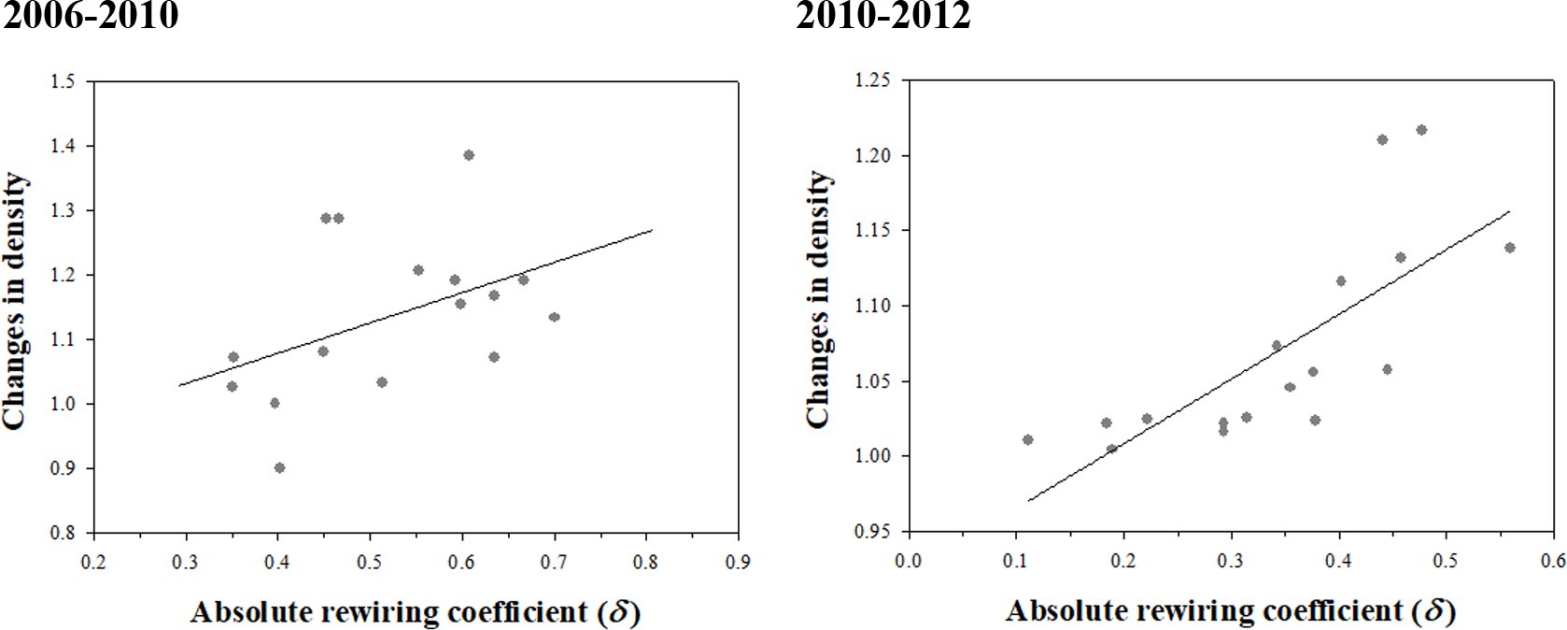
Relationship between absolute rewiring coefficients and changes of density in scientist collaboration network, 2006–2010 (left panel) and 2010–2012 (right panel).

In general, for this network our analysis again confirms that a larger amount of rewiring is associated with greater changes in network density (Fig 9). Interestingly, although the range of absolute rewiring coefficients shrank slightly, it did less so than in the case of the commuting network above. Also, the slopes of the lines in plots (right and left hand panels, Fig 9) decreased over the two time periods indicating that the relationship between the conventional and our new measure of network change also changed.

## Summary and future research

Networks adjust and change continually over time, and individual nodes may respond differently to the same shock. To understand and describe a network’s change it is critical to systematically and consistently measure the degree to which interactions among the nodes making up the network change over time. The novelty of this article is to apply a cosine distance function, as a simple measure of change, to links among nodes in a network method at two different points in time. We introduce two complementary measures, one absolute and the other relative, to measure the changes in or *rewiring* of both the entire network and of its individual components. We then apply the two methods to three different sets of network data for illustrative purposes.

As more large datasets representing networks become available, new datasets from various disciplines can be examined using the methods presented here. Also, previous studies may be updated to understand how the underlying networks have changed. From this new insights into the general dynamic behavior and adaptation of networks through rewiring may be discovered, and the effects of various degrees of rewiring can be studied. For example, in the Input-Output Table it would be of interest to assess whether the extent of economic rewiring within individual nations affected their response to the GFC, and ability to rebound. Likewise, commuting and migration network rewiring in response to changes in energy policy or home construction regulations and immigration reform could be studied over time. In turn, different degrees of rewiring may affect how quickly labor markets react to changing economic opportunities in a region or MSA, and they also may impact the amount of traffic congestion within an area. And finally, scientist citation as well as patenting networks could be investigated in terms of the impacts of changes in research funding environments, or how different levels of rewiring affect the productivity of the overall network and that of individual nodes (scientists). These are a few examples of potential new studies that could be carried out using the method proposed here.

## Supporting information

S1 File Data on the absolute and relative rewiring coefficients. (XLSX)
